# Development and validation of a risk model with variables related to non-small cell lung cancer in patients with pulmonary nodules: a retrospective study

**DOI:** 10.1186/s12885-023-11385-1

**Published:** 2023-09-18

**Authors:** Zufang Liao, Rongjiong Zheng, Ni Li, Guofeng Shao

**Affiliations:** 1 The Affiliated Lihuili Hospital, Ningbo University, Ningbo, 315041 Zhejiang China; 2 Ningbo Yinzhou NO.2 Hospital, Ningbo, 315192 Zhejiang China; 3 Department of Cardiothoracic Surgery, Li Huili Hospital Affiliated to Ningbo University, Xingning Road 57, Ningbo, 315041 Zhejiang China

**Keywords:** NSCLC, Pulmonary nodules, Logistic, Variables, Model

## Abstract

**Background:**

Lung cancer is a major global threat to public health for which a novel predictive nomogram is urgently needed. Non-small cell lung cancer (NSCLC) which accounts for the main port of lung cancer cases is attracting more and more people’s attention.

**Patients and methods:**

Here, we designed a novel predictive nomogram using a design dataset consisting of 515 pulmonary nodules, with external validation being performed using a separate dataset consisting of 140 nodules and a separate dataset consisting of 237 nodules. The selection of significant variables for inclusion in this model was achieved using a least absolute shrinkage and selection operator (LASSO) logistic regression model, after which a corresponding nomogram was developed. C-index values, calibration plots, and decision curve analyses were used to gauge the discrimination, calibration, and clinical utility, respectively, of this predictive model. Validation was then performed with the internal bootstrapping validation and external cohorts.

**Results:**

A predictive nomogram was successfully constructed incorporating hypertension status, plasma fibrinogen levels, blood urea nitrogen (BUN), density, ground-glass opacity (GGO), and pulmonary nodule size as significant variables associated with nodule status. This model exhibited good discriminative ability, with a C-index value of 0.765 (95% CI: 0.722-0.808), and was well-calibrated. In validation analyses, this model yielded C-index values of 0.892 (95% CI: 0.844-0.940) for external cohort and 0.853 (95% CI: 0.807-0.899) for external cohort 2. In the internal bootstrapping validation, C-index value could still reach 0.753. Decision curve analyses supported the clinical value of this predictive nomogram when used at a NSCLC possibility threshold of 18%.

**Conclusion:**

The nomogram constructed in this study, which incorporates hypertension status, plasma fibrinogen levels, BUN, density, GGO status, and pulmonary nodule size, was able to reliably predict NSCLC risk in this Chinese cohort of patients presenting with pulmonary nodules.

## Introduction

Lung cancer is a form of malignancy arising due to the unrestrained growth of bronchial and lung cells [1, 2], and it is one of the leading causes of mortality in the world [3]. Rates of lung cancer have been rising rapidly in recent years, particularly in more heavily industrialized nations [4]. Currently, lung cancer patients exhibit 5-year survival rates of approximately 16.6% [5], and roughly 1 million individuals in China are predicted to be diagnosed with lung cancer by the year 2025 such that China exhibits the highest global lung cancer incidence rate. Accounting for the main port of lung cancer, the early treatment of NSCLC is the hot spot of the current research.

Key risk factors associated with lung cancer development include specific genetic mutations, smoking, and environmental exposures such as air pollution. There is also some evidence suggesting that factors such as a poor diet, alcohol intake, estrogen levels, the smoking of marijuana, and infection with human papillomavirus (HPV), human immunodeficiency virus (HIV), and Epstein-Barr virus may increase lung cancer risk, although such evidence remains somewhat inconclusive [6]. Analyses of patient computed tomography (CT) scans often reveal pulmonary nodules, and many models have been developed to gauge the link between such nodules and lung cancer risk, including the Brock model [7] and the Mayo model. These models, however, often do not take epidemiological variables, clinical findings, and CT scan results into consideration at the same time, limiting their value as predictors of the relative risk of a given pulmonary nodule being malignant. The development of more reliable and accurate predictive tools has the potential to enable early intervention and treatment for NSCLC patients, maximizing their odds of positive outcomes. Herein, we analyzed 28 variables that were considered potentially relevant to the diagnosis of a given pulmonary nodule as being benign or malignant based on previous studies [1, 7–9].

By analyzing epidemiological, clinical, and CT-related factors for patients with pulmonary nodules that had undergone surgical treatment, we sought to develop a simple but robust predictive model that would enable the relative assessment of NSCLC risk based only upon characteristics that can be readily assessed prior to surgery or other therapeutic interventions.

## Materials and methods

### Patients

The Ethics Committee of the affiliated Lihuili Hospital of Ningbo University, Lihuili hospital approved this study (approval no KY2020PJ141). Enrolled patients for design cohort were individuals from China recruited at the Xingning campus of Lihuili Hospital between October 2020 to February 2022, with the external validation cohort being recruited from April 2022 to June 2022. An external validation cohort 2 was recruited from March 2023 to June 2022 at the Eastern campus of Lihuili Hospital. Eligible patients were individuals that had undergone surgical resection following pulmonary nodule identification. Small cell lung cancer cases were removed to avoid bias for the small sample size. Patients provided written informed consent to participate in this study. Inclusion criteria: (1) pulmonary nodules that were detected through CT scanning; (2) patients who were asymptomatic at time of diagnosis; (3) patients physically able to undergo surgery. Any patients diagnosed with serious cognitive or physical impairments, or other serious diseases were excluded from the study cohort. Data including patient clinical, demographic, and disease-related characteristics were retrieved from patient medical records.

### Statistical analysis

Data are given as numbers (percentages), and were analyzed using R (v 4.2.1; https://www.R-project.org) and IBM SPSS Statistics 23.0. The LASSO method, which enables the reduction of high-dimensional datasets, was utilized as a means of selecting the optimal predictors associated with NSCLC risk among the included pulmonary nodule patients. Those features that yielded non-zero coefficient values in this LASSO regression analysis were retained for nomogram incorporation. The final predictive model was constructed via a univariate logistic regression analysis followed by a multivariate logistic regression analysis, with all significance levels being two-sided. The design cohort was used to develop the predictive model, with calibration curves being used to assess nomogram calibration. Significant calibration curve results were indicative of a model that was not perfectly calibrated. Model discrimination performance was assessed based on the value of Harrell’s C-index. Validation of this nomogram was additionally performed to calculate an accurate C-index value by internal bootstrapping validation (1,000 bootstrap resamples) and external validation. Decision curve analyses were used to assess the clinical utility of this NSCLC risk nomogram by quantifying the net benefit at different probability thresholds in the cohorts, with the net benefit being calculated by subtracting the proportion of patients with false-positive results from the proportion of patients with true-positive results and by assessing the relative harm of failing to intervene as compared to the potential negative outcomes associated with an unnecessary intervention. Receiver operating characteristic (ROC) curves were also used to assess the precision of this predictive risk model. The net reclassification improvement index (NRI) and integrated discrimination improvement index (IDI) analysis were performed to calculate the improvement of the new model.

## Results

### Patient characteristics

In total, data from 515 patients with pulmonary nodules that visited our clinic between October 2020 and February 2022 were included in the design cohort for this study, while data from 140 patients collected from April 2022 to June 2022 were designed as external validation cohort and patients from Eastern campus were set as external validation cohort 2. Patients aged 21–86 (mean age: 58.97 ± 12.02 years) in the design cohort were separated into groups with benign nodules and malignant lesions, as well as patients aged 26–78 (mean age: 57.17 ± 11.18 years) in the external cohort and patients aged 22–85 (mean age: 56.52 ± 12.20 years) in the external cohort 2. For details regarding the demographic and clinical characteristics of patients in these groups, see Table 1.

**Table 1:** Baseline characteristics

Variables	Design cohort(*N* = 515)		*P*	External cohort(*N* = 140)		*P*	External cohort 2(*N* = 237)		*P*
	Benign N(%)	NSCLC N(%)		Benign N(%)	NSCLC N(%)		Benign N(%)	NSCLC N(%)	
Sex			0.217			0.658			0.060
Male	96 (53.63)	161 (47.92)		15 (38.46)	43 (42.57)		50 (49.50)	84 (61.76)	
Female	83 (46.37)	175 (52.08)		24 (51.54)	58 (57.43)		51 (50.50)	52 (38.24)	
Age(years)			0.632			0.004			0.083
= < 65	122 (68.16)	222 (66.07)		37 (94.87)	68 (67.33)		82 (81.19)	97 (71.32)	
> 65	57 (31.84)	114 (33.93)		2 (5.13)	33 (32.67)		19 (18.81)	39 (28.68)	
Education(years)			0.869			0.354			0.461
Primary (0–6)	86 (48.04)	164 (48.81)		25 (64.10)	56 (55.45)		62 (61.39)	77 (56.62)	
Higher (> 6)	93 (51.96)	172 (51.19)		14 (35.90)	45 (44.55)		39 (38.61)	59 (43.38)	
Cancer history			0.998			0.999			0.597
No	171 (95.53)	321 (95.53)		39 (100.00)	92 (91.09)		95 (94.06)	130 (95.59)	
Yes	8 (4.47)	15 (4.47)		0 (0.00)	9 (8.91)		6 (5.94)	6 (4.41)	
Smoking history			0.978			0.672			0.207
No	149 (83.24)	280 (83.33)		35 (89.74)	88 (87.13)		78 (77.23)	95 (69.85)	
Yes	30 (16.76)	56 (16.67)		4 (10.26)	13 (12.87)		23 (22.77)	41 (30.15)	
Drinking history			0.380			0.853			0.248
No	149 (83.24)	269 (80.06)		37 (94.87)	95 (94.06)		90 (89.11)	114 (83.82)	
Yes	30 (16.76)	67 (19.94)		2 (5.13)	6 (5.94)		11 (10.89)	22 (16.18)	
Hypertension statue			0.069			0.130			0.038
No	144 (80.45)	246 (73.21)		34 (87.18)	76 (75.25)		80 (79.21)	91 (66.91)	
Yes	35 (19.55)	90 (26.79)		5 (12.82)	25 (24.75)		21 (20.79)	45 (33.09)	
DM statue			0.500			0.220			0.973
No	138 (77.10)	250 (74.40)		38 (97.44)	92 (91.09)		90 (89.11)	121 (88.97)	
Yes	41 (22.90)	86 (25.60)		1 (2.56)	9 (8.91)		11 (10.89)	15 (11.03)	
BMI (Kg/m^2^)			0.217			0.268			0.372
Normal (18.5 ≤ *X* < 25)	119 (66.48)	241 (71.73)		16 (41.03)	52 (51.49)		65 (64.36)	95 (69.85)	
Abnormal (others)	60 (33.52)	95 (28.27)		23 (58.97)	49 (48.51)		36 (35.64)	41 (30.15)	
Location									
Left lower lobe	30 (16.76)	57 (16.97)	0.561	11 (28.21)	16 (15.84)	0.257	21 (20.79)	21 (15.44)	0.131
Other lobes	95 (53.07)	163 (48.51)	0.695	16 (41.03)	50 (49.51)	0.115	57 (56.44)	68 (50.00)	0.621
Right upper lobe	54 (30.17)	116 (34.52)	0.660	12 (30.76)	35 (34.65)	0.177	23 (22.77)	47 (34.56)	0.074
Border clear			0.230			0.425			0.000
No	27 (15.08)	65 (19.35)		1 (2.56)	6 (5.94)		7 (6.93)	41 (30.15)	
Yes	152 (84.92)	271 (80.65)		38 (97.44)	95 (94.06)		94 (93.07)	95 (69.85)	
Spicule sign			0.977			0.102			0.000
No	157 (87.71)	295 (87.80)		38 (97.44)	88 (87.13)		94 (93.33)	96 (70.59)	
Yes	22 (12.29)	41 (12.20)		1 (2.56)	13 (12.87)		7 (6.67)	40 (29.41)	
Density									
High	117 (65.36)	110 (32.74)	0.000	36 (92.31)	34 (33.66)	0.000	48 (47.52)	43 (31.62)	0.000
Low	40 (22.35)	146 (43.45)	0.000	3 (7.69)	47 (46.54)	0.000	49 (48.52)	25 (18.38)	0.082
Mediate	22 (12.29)	80 (23.81)	0.000	0 (0.00)	20 (19.80)	0.998	4 (3.96)	68 (50.00)	0.000
GGO			0.000			0.000			0.000
No	142 (79.33)	147 (43.75)		37 (94.87)	40 (39.60)		66 (65.35)	33 (24.26)	
Yes	37 (20.67)	189 (56.25)		2 (5.13)	61 (60.40)		35 (34.65)	103 (75.74)	
Size			0.023			0.006			0.000
< 8 mm	28 (15.64)	30 (8.93)		12 (30.77)	11 (10.89)		29 (28.71)	9 (6.62)	
≥ 8 mm	151 (84.36)	306 (91.07)		27 (69.23)	90 (89.11)		72 (71.29)	127 (93.38)	
Vessels pass through			0.036			0.999			0.000
No	167 (93.30)	293 (87.20)		39 (100.00)	97 (96.04)		89 (88.12)	82 (60.29)	
Yes	12 (6.70)	43 (12.80)		0 (0.00)	4 (3.96)		12 (11.88)	54 (39.71)	
INR			0.988			0.760			0.596
Normal (1.6–2.0)	170 (94.97)	319 (94.94)		37 (94.87)	97 (96.04)		94 (93.07)	124 (91.18)	
Abnormal (others)	19 (5.03)	17 (5.06)		2 (5.13)	4 (3.96)		7 (6.93)	12 (8.82)	
Plasma fibrinogen level			0.000			0.767			0.045
Normal (2-4 g/L)	155 (86.59)	240 (71.43)		28 (71.79)	75 (74.26)		89 (88.12)	106 (77.94)	
Abnormal (others)	24 (13.41)	96 (28.57)		11 (28.21)	26 (25.74)		12 (11.88)	30 (22.06)	
Plasma albumin			0.471			0.754			0.011
Normal (40-55 g/L)	96 (53.63)	169 (50.30)		22 (56.41)	54 (53.47)		90 (89.11)	103 (75.74)	
Abnormal (others)	83 (46.37)	167 (49.70)		17 (43.59)	47 (46.53)		11 (10.89)	33 (24.26)	
GGT			0.452			0.338			0.889
Normal (7-45U/L)	157 (87.71)	302 (89.88)		38 (97.44)	94 (93.07)		87 (86.14)	118 (86.76)	
Abnormal (others)	22 (12.29)	34 (10.12)		1 (2.56)	7 (6.93)		14 (13.86)	18 (13.24)	
Blood glucose (mmol/L)			0.472			0.817			0.542
Normal (3.89–6.11)	150 (83.80)	273 (81.25)		33 (84.62)	87 (86.14)		82 (81.19)	106 (77.94)	
Abnormal (others)	29 (16.20)	63 (18.75)		6 (15.38)	14 (13.86)		19 (18.81)	30 (22.06)	
CR			0.982			0.148			0.930
Normal	161 (89.94)	302 (89.88)		38 (97.44)	90 (89.11)		91 (90.00)	123 (90.44)	
Abnormal (others)	18 (10.06)	34 (10.12)		1 (2.56)	11 (10.89)		10 (10.00)	13 (9.56)	
BUN			0.023			0.148			0.803
Normal	173 (96.65)	306 (91.07)		38 (97.44)	90 (89.11)		93 (90.10)	124 (91.18)	
Abnormal (others)	6 (3.35)	30 (8.93)		1 (2.56)	11 (10.89)		8 (9.90)	12 (8.82)	
SUA			0.047			0.906			0.283
Normal	155 (86.59)	267 (79.46)		32 (82.05)	82 (81.19)		75 (74.26)	109 (80.15)	
Abnormal (others)	24 (13.41)	69 (20.54)		7 (17.95)	19 (18.81)		26 (25.74)	27 (19.85)	
TG (mmol/L)			0.037			0.534			0.596
Normal (0.56–1.7)	130 (72.63)	271 (80.66)		32 (82.05)	78 (77.23)		65 (64.36)	92 (67.65)	
Abnormal (others)	49 (27.37)	65 (19.34)		7 (17.95)	23 (22.77)		36 (35.64)	44 (32.35)	
TC (mmol/L)			0.750			0.672			0.933
Normal (2.84–5.69)	145 (81.01)	276 (82.14)		35 (89.74)	88 (87.13)		85 (84.16)	115 (84.56)	
Abnormal (others)	34 (18.99)	60 (17.86)		4 (10.26)	13 (12.87)		21 (15.84)	21 (15.44)	
HDL (mmol/L)			0.295			0.060			0.487
Normal (1.03–1.55)	115 (64.25)	200 (59.52)		27 (69.23)	52 (51.49)		61 (60.40)	76 (55.88)	
Abnormal (others)	64 (35.75)	136 (40.48)		12 (30.77)	49 (48.51)		40 (39.60)	60 (44.12)	
LDL (mmol/L)			0.569			0.787			0.416
Normal (1.55–3.36)	154 (86.03)	295 (87.80)		35 (89.74)	89 (88.12)		81 (80.20)	103 (75.74)	
Abnormal (others)	25 (13.97)	41 (12.20)		4 (10.26)	12 (11.88)		20 (19.80)	33 (24.26)	

“Normal” standard for “CR”, “BUN” and “SUA”: CR (man: 57-111 μmol/L; woman: 41–61 μmol/L), BUN (man: 3.6–9.5 mmol/L; woman: 3.1–8.8 mmol/L), SUA (man: 210-420 μmol/L; woman: 150–350 μmol/L)

*GGT* gamma-glutamyl transpeptidase, *CR* Creatinine, *BUN* Blood urea nitrogen, *SUA* Serum uric acid

### Feature selection and predictive model development

**Fig. 1:**
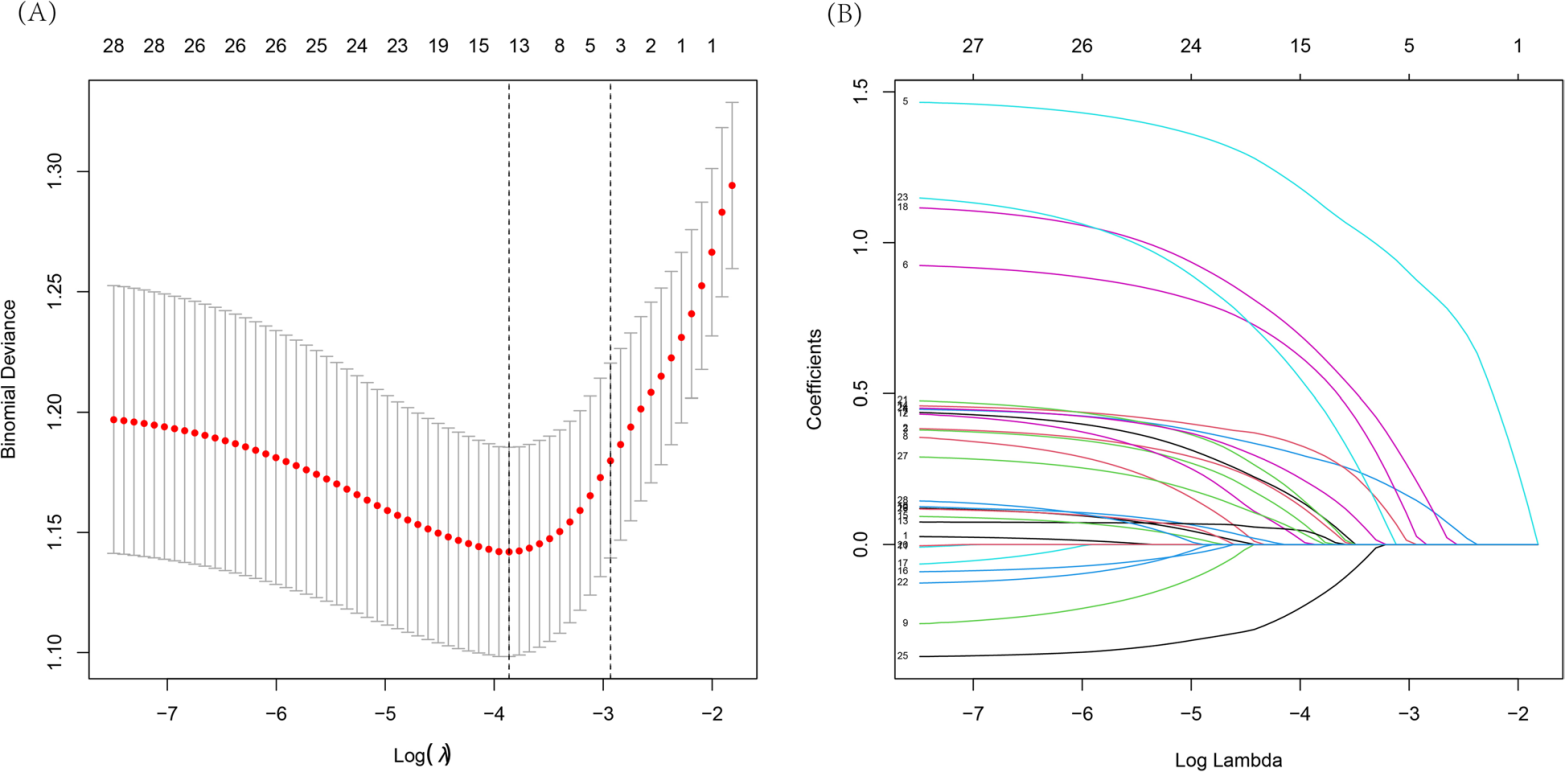
LASSO binary logistic regression model-based clinicopathological feature selection. Notes: **A** Five-fold cross-validation was used for optimal parameter (λ) selection in the LASSO model via minimum criteria, with a partial likelihood deviance (binomial deviance) curve being plotted against log(λ). Optimal values were marked with dashed vertical lines at optimal values using minimum criteria with the 1-SE criteria. The selected optimal λ value was 0.021. **B** LASSO coefficient profiles for 28 potential features were generated, with coefficient profile plots against the log(lambda) sequence being generated. Five-fold validation was used to draw vertical lines at selected values, with optimal lambda results yielding fourteen total features with non-zero coefficient values. SE: standard error

**Fig. 2:**
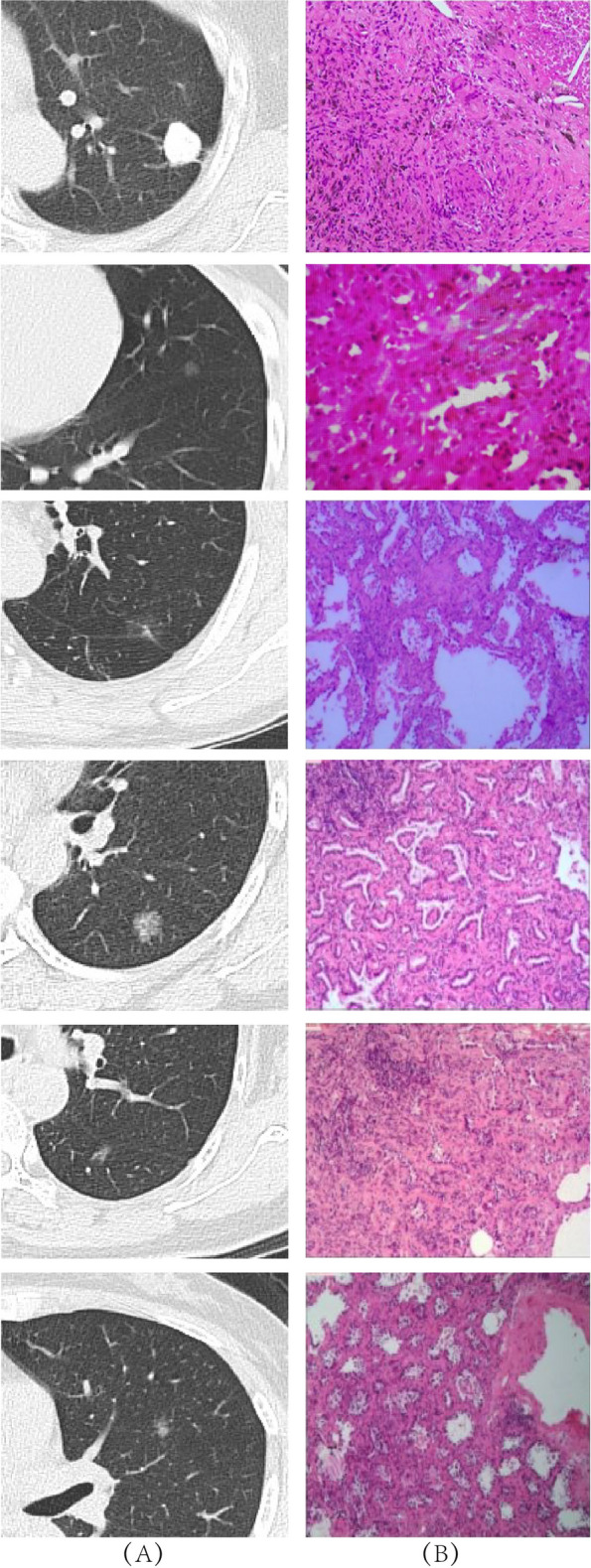
Different lung nodules represent their characteristics in CT scan (**A**) and correlative pathology stained by hematoxylin & eosin (**B**). Row 1: the pathological finding of a 16-mm high-density nodule for a 52-year-old woman with is benign (X 100); Row 2: the pathological finding of a 5.7-mm low-density nodule for a 34-year-old woman was a carcinoma in situ (X 200); Row 3: the pathological finding of a 8-mm low-density nodule for a 56-year-old man was a MIA (X 100); Row 4: the pathological finding of an 11-mm low-density nodule for a 50-year-old woman was an IAC (X 100); Row 5: the pathological finding of a 7-mm partly solid-density nodule for a 63-year-old woman was a MIA (X 100); Row 6: the pathological finding of an 11-mm partly solid-density nodule for a 81-year-old woman was an IAC (X 100)

**Fig. 3:**
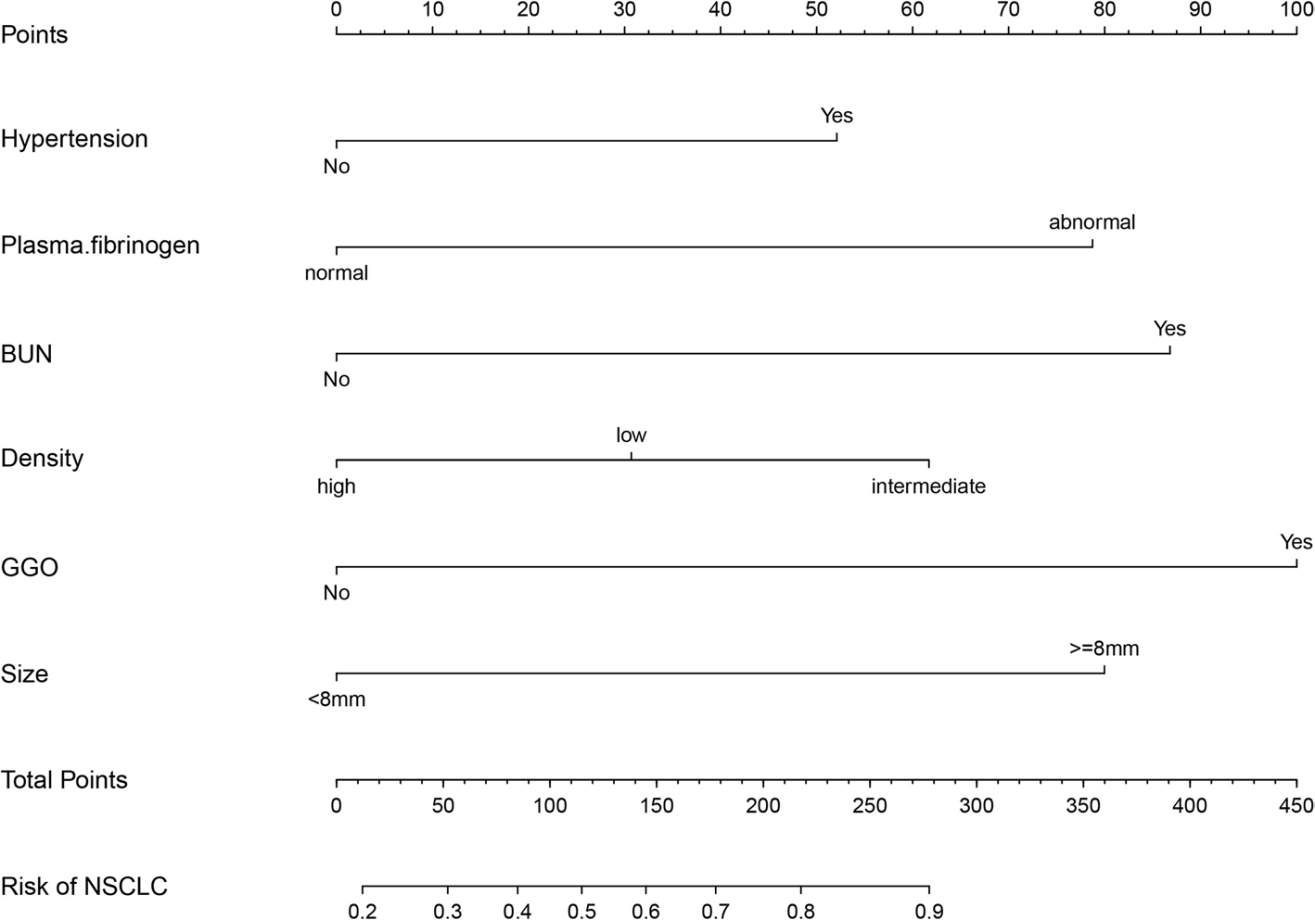
NSCLC risk nomogram. Note: An initial design cohort was used to develop this nomogram, which incorporated hypertension, BUN, plasma fibrinogen, pulmonary nodule size, GGO status, density. BUN: blood urea nitrogen. GGO: ground-glass opacity

In total, 28 potentially relevant features were evaluated for inclusion in a predictive model. Of these features, 14 were ultimately selected through a LASSO regression analysis of the 515 patients in the design cohort (Fig. 1A and B). These features included “border clear”, vessels pass through, hypertension status, smoking history, drinking history, blood glucose, BUN, serum uric acid (SUA), triglyceride (TG) levels, plasma fibrinogen levels, density, ground-glass opacities (GGOs), spicule sign, and pulmonary nodule size. Then, in Table 2, univariate and multivariate logistic regression analyses were performed. The *P*-value of 0.624 in the Hosmer–Lemeshow test indicated non-significance. CT characteristics and correlative pathological results of representative nodules were shown in Fig. 2. A predictive model incorporating these significant variables was developed using the design cohort (Fig. 3). Nodule density was defined as being “low” when it exhibited a CT value that was higher than that of pulmonary tissue but lower than that of pulmonary vessels, “intermediate” for nodules with solid and GGO components, and “high” when CT values were greater for the nodule than for pulmonary vessels. While the features of Mayo model were smoking history, age, nodule diameter, cancer history, site in the left and spicule sign. This model was explained by the calculation formula: P = e^*x*^/(1 + e^*x*^), where *x* =  − 6.8272 + (0.7917 × smoking history) + (0.0391 × age) + (0.1274 × nodule diameter) + (1.3388 × cancer history) + (0.7838 × the upper lobe) + (1.0407 × spicule sign). One recent research [10] which was published in *Chest* showed that the parsimonious Brock model (including gender, size, upper location and spicule sign) could predict cancer risk well, and we calculated the performance of the model in our cohorts.

**Table 2:** The features for patients with pulmonary nodules in the design cohort using multivariate logistic regression analyses

**Variables**		**Univariate**		**Multivariate**	
		**OR (95% CI)**	***P* value**	**OR (95% CI)**	***P* value**
Hypertension	yes	1.505(0.968–2.340)	0.069	1.892(1.150–3.111)	0.012
Plasma fibrinogen	abnormal	2.583(1.581–4.220)	0.000	2.731(1.582–4.713)	0.000
BUN	abnormal	2.827(1.154–6.926)	0.023	3.093(1.169–8.182)	0.023
SUA	abnormal	1.669(1.007–2.765)	0.047	-	-
TG	abnormal	1.571(1.027–2.405)	0.037	-	-
Density	high	-	0.000	-	0.039
	intermediate	3.868(2.256–6.630)	0.000	2.304(1.210–4.389)	0.011
	low	3.882(2.510–6.004)	0.000	-	0.261
GGO	yes	4.934(3.239–7.518)	0.000	3.866(1.957–7.637)	0.000
Size	≥ 8 mm	1.891(1.091–3.280)	0.023	2.879(1.497–5.538)	0.002
Vessels pass through	yes	2.04(1.05–3.98)	0.036	-	-

We classified GGOs as pure GGOs (pGGOs, *n* = 208) and mixed GGOs (mGGOs, *n* = 81). The relationship between them and lung cancers were further analyzed. *P*-value of 0.460 was got in univariate analysis and NSCLC was excluded in the forward likelihood ratio logistic analyses (Table 3). Further, mGGOs was positively correlated with nodule size when compared with pGGOs.

**Table 3:** Variables significance analyses in the two cohorts using multivariate logistic regression by forward stepwise likelihood ratio way

**Variables**		**Univariate**		**Multivariate**	
		**OR (95% CI)**	***P* value**	**OR (95% CI)**	***P* value**
NSCLC	yes	1.35(0.61–2.98)	0.460	-	-
Vessels pass through	yes	2.02(0.94–4.34)	0.072	-	-
Size	≥ 8 mm	4.44(1.53–12.87)	0.006	4.49(1.55–13.08)	0.006
Education	primary	1.76(1.05–2.95)	0.033	-	-
BMI	abnormal	0.54(0.29–0.98)	0.044	0.53(0.29–0.98)	0.042
GGT	abnormal	0.34(0.10–1.18)	0.090	-	-

*BMI* Body Mass Index, *GGT* Gamma-glutamyl transpeptidase

### Assessment of predictive risk model performance

**Fig. 4:**
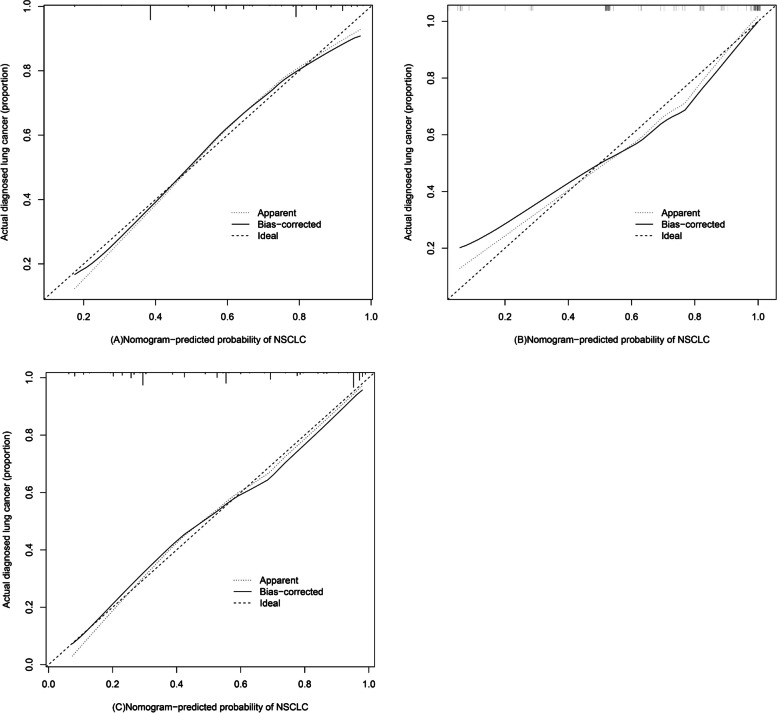
Calibration curves for NSCLC nomogram predictions in the design cohort (**A**), external validation cohort (**B**) and external validation cohort 2 (**C**). Note: Predicted risk of NSCLC and actual NSCLC diagnoses are shown on the x-axis and y-axis, respectively, with the dotted line corresponding to a diagnostic model with perfect predictive accuracy and the solid line corresponding to actual nomogram performance. The closer these lines are to one another, the better the predictive performance of this nomogram

Calibration curves for this predictive nomogram when used to analyze the design cohort revealed it to be well-calibrated, with a C-index value of 0.765 (95% CI: 0.722–0.808) (Fig. 4A). Similarly, the C-index values for the external validation cohort, external validation cohort 2 and internal bootstrapping validation were 0.892 (95% CI: 0.844–0.940) (Fig. 4B), 0.853 (95% CI: 0.807–0.899) (Fig. 4C) and 0.753, respectively, consistent with the discriminative value of this model, suggesting that it exhibits good predictive utility.

### Ten-fold cross-validation analyses

Ten-fold cross-validation analyses were performed in the two cohorts (Table 4). As the sample size of validation cohort (Tables 5 and 6) was small, we resampled the cohort at 50 times. Our model showed good stabilities for its well kappa results. The model predicted accurately for the good AUC and Accuracy value (design cohort [AUC = 0.747 ± 0.081; Accuracy = 0.732 ± 0.064; Kappa = 0.376 ± 0.155] *vs.* validation cohort [AUC = 0.849 ± 0.104; Accuracy = 0.761 ± 0.092; Kappa = 0.321 ± 0.259] *vs.* validation cohort 2 [AUC = 0.833 ± 0.079; Accuracy = 0.737 ± 0.087; Kappa = 0.461 ± 0.179]).

**Table 4:** Ten-fold cross-validation analysis for the design cohort

Group	1	2	3	4	5	6	7	8	9	10
Kappa	0.400	0.548	0.337	0.283	0.235	0.457	0.477	0.628	0.283	0.116
Accuracy	0.745	0.804	0.712	0.692	0.654	0.765	0.770	0.843	0.692	0.647
AUC	0.798	0.854	0.753	0.767	0.613	0.691	0.806	0.835	0.641	0.719

The values of kappa, accuracy and AUC in the design cohort are shown in the table. The final average is calculated from ten groups (AUC = 0.747 ± 0.081; Accuracy = 0.732 ± 0.064; Kappa = 0.376 ± 0.155)

**Table 5:** Ten-fold cross-validation analysis for the validation cohort

Group	1	2	3	4	5	6	7	8	9	10
Kappa	0.435	0.176	0.323	0.432	0.000	0.391	0.588	0.364	0.322	0.189
Accuracy	0.714	0.643	0.857	0.714	0.857	0.786	0.857	0.786	0.857	0.786
AUC	0.803	0.775	0.963	0.675	0.913	0.900	0.950	0.825	0.725	0.888

The values of kappa, accuracy and AUC in the validation cohort are shown in the table. The final average is calculated from ten groups (AUC = 0.849 ± 0.104; Accuracy = 0.761 ± 0.092; Kappa = 0.321 ± 0.259)

**Table 6:** Ten-fold cross-validation of the model in the validation cohort 2

Group	1	2	3	4	5	6	7	8	9	10
Kappa	0.563	0.667	0.565	0.232	0.374	0.276	0.746	0.333	0.401	0.565
Accuracy	0.783	0.833	0.792	0.640	0.696	0.652	0.875	0.667	0.696	0.792
AUC	0.800	0.900	0.868	0.779	0.754	0.723	0.929	0.846	0.777	0.896

The values of kappa, accuracy and AUC in the validation cohort are shown in the table. The final average from ten groups are calculated (Kappa = 0.461 ± 0.179; Accuracy = 0.737 ± 0.087; AUC value = 0.833 ± 0.079)

### Different types of NSCLC compared with normal cases by multinomial logistic analyses

We classified NSCLC into carcinoma in situ, minimally invasive adenocarcinoma (MIA), invasive adenocarcinoma (IAC) and other types according to pathological results. MIA and IAC accounted for 80.32%, and the rest types of NSCLC accounted for only 19.68%. Among them, for example, the degrees of invasion between minimally invasive adenocarcinoma (MIA) and invasive adenocarcinoma (IAC) are incremental. Therefore, the assessment of each factor in the model among the various types of NSCLC was necessary. We excepted carcinoma in situ for the small sample size and mainly concentrated on evaluating the associations with MIA and IAC in different factors using binomial and multinomial logistic regression analyses. The features in the model were related with them (Table 7): GGO (odds ratio [OR] 7.13 [95% CI, 3.25–19.63] and 3.03 [95% CI, 1.47–6.25]) in MIA and IAC, density (OR 5.79 [95% CI, 2.46–13.65] and 2.53 [95% CI, 1.28–5.02]) in MIA and IAC, and nodule size (OR 2.58 [95% CI, 1.20–5.54]; 6.51 [95% CI, 2.71- 15.61] and 5.98 [95% CI, 1.38–25.83]) in MIA, IAC and other types. Risk of MIA and IAC in intermediate-density lung nodules was of significance when compared with high-density nodules, moreover, the risk of IAC was only a half of that of IAC. The analysis of GGO shown a similar trend. Sizes of nodules in different types NSCLC were all significant (*P*:0.015 *vs.* 0.000 *vs.* 0.017), what’s more, when the pulmonary nodule size was ≥ 8 mm the degree of infiltration might be deeper in MIA and IAC. Except for the above features, IAC and other types in the multinomial models had a same risk factor hypertension, while BUN and plasma fibrinogen levels seemed to be risk factors of MIA.

**Table 7:** Odds ratios of model variables in different types of NSCLC compared with normal cases using binomial and multinomial logistic regression analyses

**Variables**	**MIA (*n* = 160)**				**IAC (*n* = 187)**				**Other (*n* = 54)**			
	**Univariable** **OR (95% CI)**	***P***	**Multivariable OR (95% CI)**	***P***	**Univariable** **OR (95% CI)**	***P***	**Multivariable OR (95% CI)**	***P***	**Univariable** **OR (95% CI)**	***P***	**Multivariable OR (95% CI)**	***P***
Density	-	0.000	-	-	-	0.000	-	-	-	0.699	-	-
Low	19.47(10.62–35.70)	0.000	3.97(1.60–9.85)	0.003	2.74(1.70–4.40)	0.000	1.32(0.61–2.83)	0.480	0.80(0.36–1.78)	0.586	1.33(0.45–3.95)	0.610
Intermediate	17.44(8.67–35.09)	0.000	5.79(2.46–13.65)	0.000	4.01(2.28–7.07)	0.000	2.53(1.28–5.02)	0.008	0.67(0.22–2.03)	0.475	0.81(0.25–2.60)	0.718
GGO (yes)	19.04(11.32–32.03)	0.000	7.13(3.25–15.63)	0.000	3.73(2.39–5.84)	0.000	3.03(1.47–6.25)	0.003	0.57(0.23–1.43)	0.572	0.53(0.15–1.85)	0.319
Size (≥ 8 mm)	1.72(0.95–3.14)	0.074	2.58(1.20–5.54)	0.015	5.62(2.45–12.88)	0.000	6.51(2.71–15.61)	0.000	5.68(1.33–24.30)	0.019	5.98(1.38–25.83)	0.017
Hypertension (has)	0.86(0.50–1.48)	0.588	1.03(0.54–1.96)	0.919	2.48(1.58–3.89)	0.000	2.85(1.74–4.66)	0.000	2.22(1.15–4.29)	0.018	2.23(1.14–4.36)	0.019
PF (abnormal)	2.65(1.63–4.30)	0.000	2.77(1.54–4.97)	0.001	1.74(1.07–2.84)	0.025	2.03(1.19–3.46)	0.009	1.33(0.63–2.82)	0.459	1.49(0.69–3.23)	0.308
BUN (abnormal)	3.62(1.54–8.50)	0.003	4.49(1.64–12.27)	0.003	2.34(0.97–5.66)	0.058	2.39(0.92–6.21)	0.075	1.58(0.41–6.17)	0.510	1.64(0.41–6.60)	0.483

*PF* abnormal plasma fibrinogen levels, *Other* other types of NSCLC, *BUN* abnormal blood urea nitrogen

### Analysis of model clinical utility

**Fig. 5:**
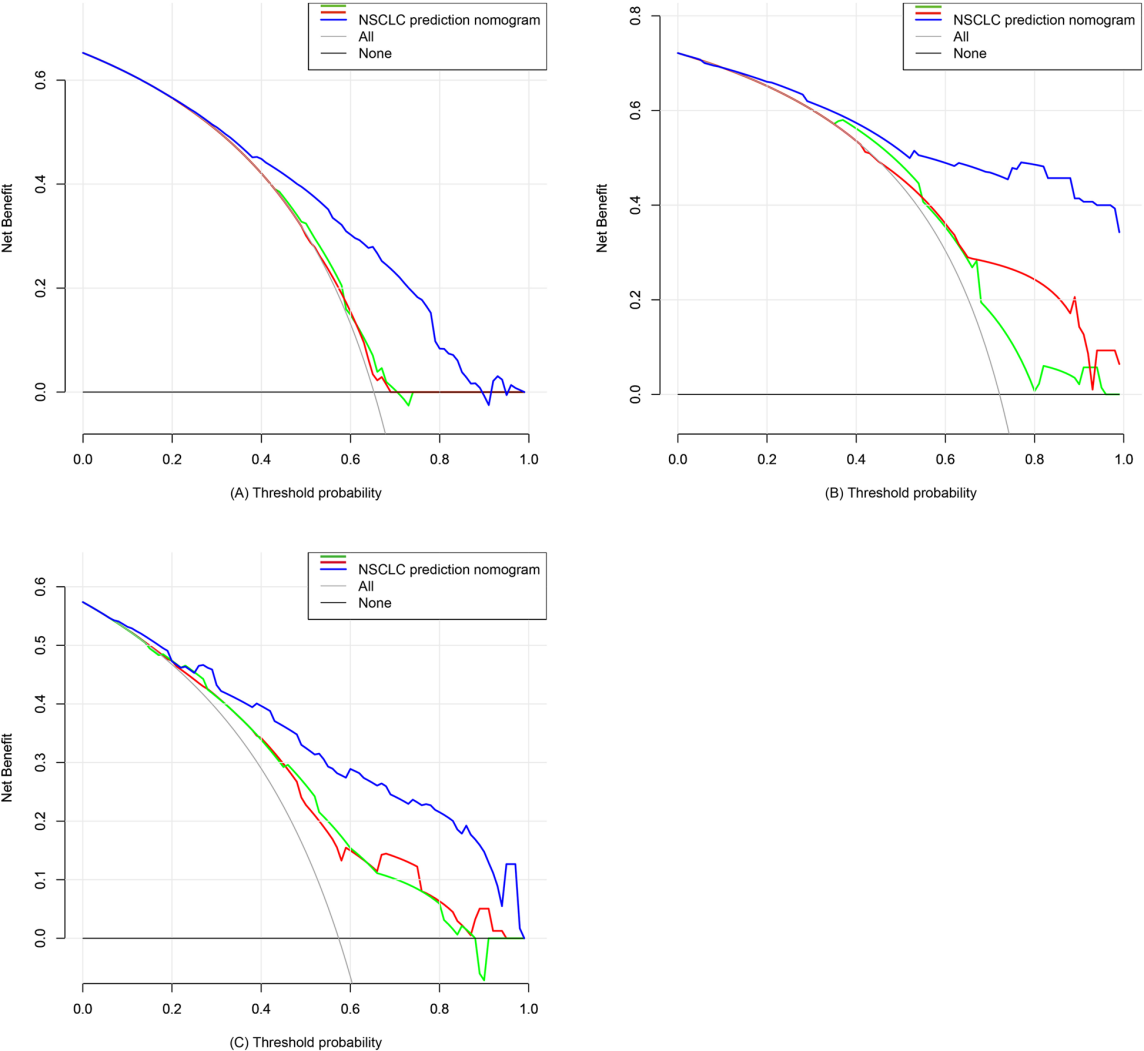
Decision curve analysis. Notes: Net benefit is shown on the y-axis, with the blue or red line corresponding to the NSCLC risk nomogram. The thin and thick lines respectively correspond to the assumptions that all patients or no patients got NSCLC, with the decision curve demonstrating that if the threshold probability of a patient and a doctor is > 18% and < 90% (**A**), > 3% (**B**) and > 7% and < 90% (**C**) in our model for the three cohorts, respectively, then the use of this nomogram to predict the risk of NSCLC is more beneficial than a treat-all or treat-none interventional scheme for these patients. The red line stands for Mayo model, the blue line stands for our model and the green line stands for parsimonious version of the Brock model

Decision curve analyses for this predictive nomogram were next performed (Fig. 5). These analyses revealed that at a threshold probability of a patient and a doctor is > 18 and < 90% and > 3% in the two cohorts, respectively, then this nomogram exhibits value as a means of predicting NSCLC risk. Net benefit was comparable with some overlap within this range when assessing NSCLC risk based on this nomogram. Our model (the blue line) showed a higher overall net benefit (Fig. 5A, B, C) when compared with the Mayo models (the red line) and simplified Brock model (the green line) in the two cohorts.

### ROC curve analysis

**Fig. 6:**
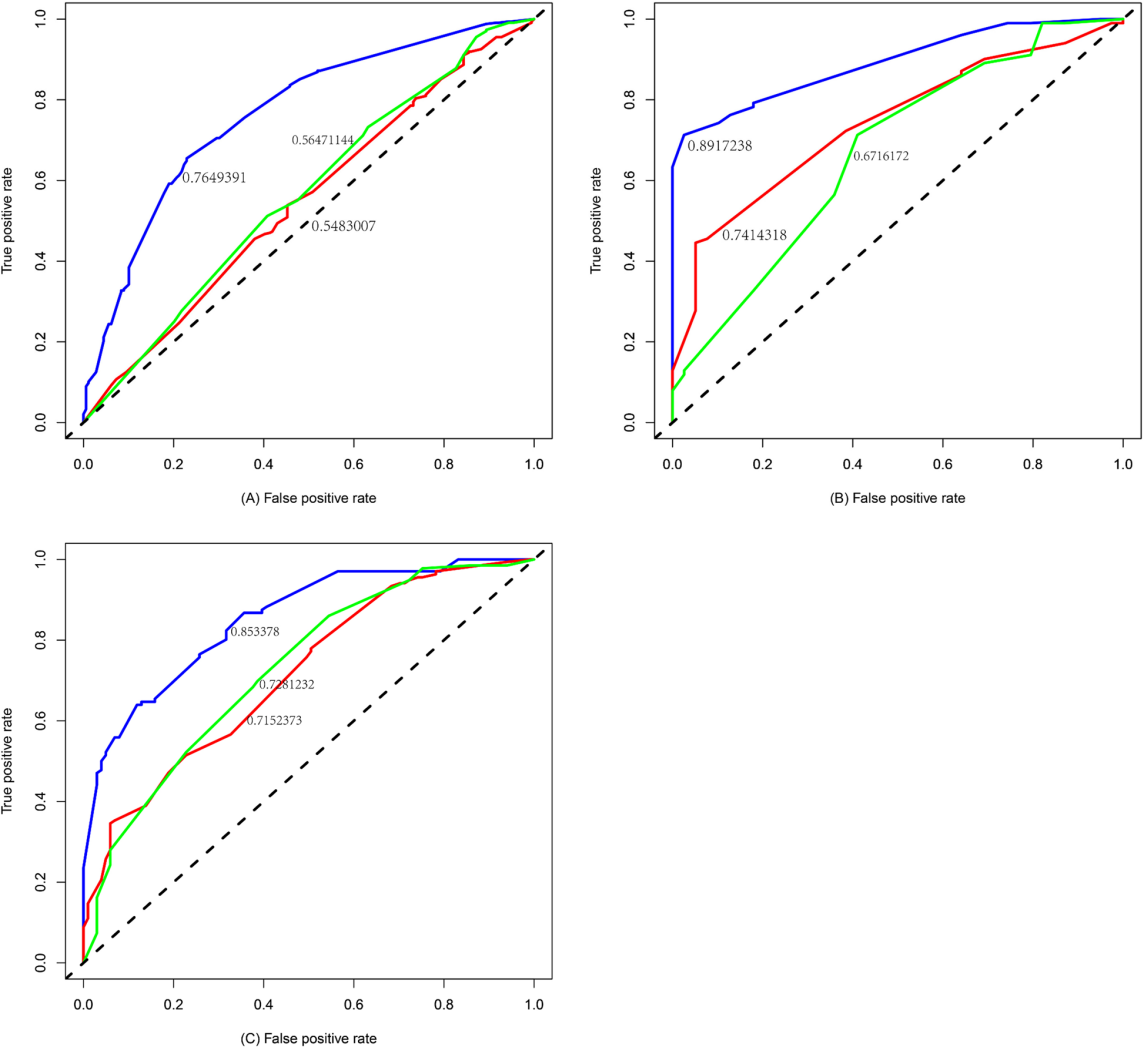
Receiver operating characteristic curve analyses for the design cohort (**A**), external validation cohort (**B**) and external validation cohort 2 (**C**). The red line stands for Mayo model, the blue line stands for our model and the green line stands for parsimonious version of the Brock model

ROC curve analyses of the two cohorts included in this study confirmed the predictive value of the two model, with an area under the curve value of 0.765 *vs.* 0.548 *vs.* 0.565 for the design cohort (Fig. 6A) and 0.892 *vs.* 0.741 *vs.* 0.672 for the external validation cohort (Fig. 6B) and 0.853 *vs.* 0.715 *vs.* 0.728 for the external validation cohort (Fig. 6C). The adopting the area under the ROC curve (AUC) values of our model (the blue line) were all higher than that of Mayo model (the red line) and parsimonious version of the Brock model (the green line).

### NRI and IDI analysis of the three models

As a supplement for the comparison of the AUC values, we calculated the net reclassification improvement index (NRI) and integrated discrimination improvement index (IDI) of the two models to research the improvement of our model (Table 8). When compared our model with the two models in design cohort, the NRI and IDI were [Mayo: 37.41 (95%CI: 0.29–0.46,* P* = 0.000) and Brock:32.49 (95%CI: 0.23–0.42,* P* = 0.000)] and [Mayo: 18.53 (95%CI: 0.15–0.22,* P* = 0.000) and Brock:17.49(95%CI: 0.14–0.21,* P* = 0.000)], respectively. In the external validation cohort, the NRI and IDI were [Mayo:34.15 (95%CI: 0.15–0.53,* P* = 0.000) and Brock:25.23(95%CI: 0.07–0.43, *P* = 0.006)] and [Mayo:26.86 (95%CI: 0.18–0.35,* P* = 0.000) and Brock:32.25(95%CI: 0.24–0.41, *P* = 0.000)], respectively. In the external validation cohort2, the NRI and IDI were [Mayo: 20.28 (95%CI: 0.03–0.38,* P* = 0.021) and Brock:24.08(95%CI: 0.08–0.40,* P* = 0.003)] and [Mayo: 19.61 (95%CI: 0.13–0.26,* P* = 0.000) and Brock:19.72(95%CI: 0.13–0.26,* P* = 0.000)], respectively. All the *P* values of them were of significance, meaning that our model could identify the benign and malignant nodules more accurately.

**Table 8:** The analysis of NRI and IDI for the design cohort, external cohort and external cohort 2 were used to assess reclassification performance and improvement in discrimination of our model

	NRI			IDI		
	%	95%CI	***P* value**	%	95%CI	***P* value**
Design cohort						
Ours vs. Mayo	37.41	0.29–0.46	0.000	18.53	0.15–0.22	0.000
Design cohort						
Ours vs. Brock	32.49	0.23–0.42	0.000	17.49	0.14–0.21	0.000
External cohort						
Ours vs. Mayo	34.15	0.15–0.53	0.000	26.86	0.18–0.35	0.000
External cohort						
Ours vs. Brock	25.23	0.07–0.43	0.006	32.25	0.24–0.41	0.000
External cohort 2						
Ours vs. Mayo	20.28	0.03–0.38	0.021	19.61	0.13–0.26	0.000
External cohort 2						
Ours vs. Brock	24.08	0.08–0.40	0.003	19.72	0.13–0.26	0.000

## Discussion

Nomograms are valuable predictive tools that have been widely utilized in oncology and other clinical and research fields, offering a user-friendly approach to intuitively assessing the odds of a given diagnosis or outcome based on a set of specific variables, thereby aiding in clinical decision-making [11]. Many models for the treatment of pulmonary nodules were established based upon certain epidemiological variables and CT scan results. However, clinical findings such as hematological biomarkers are also very important for the diagnosis of lung cancer [1]. Moreover, for some of these variables, such as GGO, the surgical criteria are not well defined such that treatments are often conducted according to the experience of the operating surgeons [12, 13]. As such, we herein sought to develop a new nomogram capable of predicting the relative risk of malignancy when evaluating patients with pulmonary nodules.

We designed and validated a novel predictive model capable of assessing the risk of a given lung nodule being benign or malignant based on analysis of data from patients that had undergone pulmonary nodule resection. The resultant model incorporated demographic, disease-, and treatment-related features to easily predict the odds of a given pulmonary nodule corresponding to a NSCLC diagnosis. The model developed herein was accurate, and exhibited good calibration and discrimination in our validation cohort. The C-index value in this validation cohort was also high, indicating that the nomogram can be accurately used to gauge patient risk of pulmonary nodule malignancy.

Prior studies have confirmed that hypertension is a common comorbidity in cancer patients [14]. Several mechanisms may explain this observation, including the fact that hypertension can increase VEGF levels in the plasma [15]. We identified hypertension as a risk factor for lung nodule malignancy. Fibrinogen has also been significantly linked to the risk of lung cancer in the past [16], with Kuang et al. having demonstrated that a combination of the beta and gamma chains of fibrinogen may offer value as a sensitive biomarker for differentiating between lung nodules that are benign and malignant [17], potentially explaining the significance of plasma fibrinogen levels in our model. One research indicated that the value of BUN to seralbumin ratio might predict patients with serious pulmonary cancer [18]. BUN had a positive relationship with pulmonary tumor risk and was included in risk prediction model therefore [19]. Some researches demonstrated that the maximum diameter of nodules > 8 mm was independent risk factors for malignancy [20] and presence of solid element in the GGO nodules might cause lymph node metastasis [21]. GGO findings have been reported to be associated with cancer rates as high as 63%, with many surgeons believing that GGO nodules should be resected, particularly if they grow in size. Persistent GGO nodules may be indicative of a greater risk of malignancy when solid components are evident [12]. Tu et al. found CT density to be a valuable feature when differentiating between nodules that were malignant and benign [22]. Qiu et al. further determined that solitary ground-glass opacity nodule size and density upon high-resolution T evaluation were associated with invasive adenocarcinoma risk [23]. Nodule size may be the most important variable included in our predictive model, given that nodule diameter is a key determinant of treatment under the British Thoracic Society guidelines [24] and Fleischner Society Guidelines [25]. For nodules ≥ 10 mm in diameter, the odds of malignancy in the NELSON screening study were 15.2% [26]. As such, we included nodule diameter as the size variable in the present study. As the comparison of the AUC value between different models had certain limitations, we calculated the NRI and IDI of the two models to explain the improvement of our model.

Herein, we thus designed a risk nomogram that may aid clinicians in differentiating between patients with benign or malignant lung nodules. It may also aid in the optimal selection of pulmonary nodules in the context of clinical research. For example, this model might be used to aid investigators in selecting patients with larger nodules and other risk-related findings when identifying candidates for surgical procedures or other interventions. Early interventions including CT scans, biochemical analyses of blood samples, and family support can better benefit low-risk patients, while regular clinical examination can ensure the appropriate monitoring of lung nodules to better guide the appropriate assessment of patient diagnosis.

Previous classical models based on large-scale screening experiments have been widely used for clinical evaluation. However, people who go to different hospitals for treatment are inevitably screened by human factors. For example, as a tertiary hospital, our hospital serves for many patients come from subordinate hospitals, which may express the high proportion of patients with ≥ 8 mm and malignant nodules in our cohorts. Therefore, it is necessary to develop clinical assessment models for pulmonary nodules based on different groups of patients. Accurate predictive evaluation can aid surgeons in predicting lung cancer risk in individual patients, ensuring timely intervention for high-risk patients while reducing the need for interventional treatment in low-risk patients. Accurately predicting the risk of lung cancer in a given patient is very challenging, and appropriate measurements together with multifaceted interventional approaches are thus the most reliable approach to detecting and evaluating patients with pulmonary nodules. Further research on this topic is warranted as the accurate detection of pulmonary nodules alone is necessary but insufficient for treating affected patients, underscoring directions for future study.

Although our model showed good accuracy and stability in different validation cohorts. Among the variables included in the model, BUN demonstrated statistical significance solely within the training cohort, while it did not exhibit significance in both the external validation cohort and external validation cohort 2. This suggests potential instability of this index and highlights room for improvement within the model. The presence of these findings indicates that there is still scope for enhancing the current study's model, which is currently limited by its inclusion of a restricted number of variables. With the continuous advancement of artificial intelligence technology, we believe that future research endeavors will benefit from larger training cohorts encompassing more diverse variables, thereby facilitating the establishment of more precise and straightforward prediction models.

### Limitations

There are multiple limitations to this study. For one, all patients in our study were enrolled from a single center over a relatively limited study period. Additionally, risk factor analyses did not incorporate all possible risk factors that may be relevant to the differentiation between benign and malignant nodules. Other relevant factors not included in this analysis included the number of nodules and specific comorbidity incidence rates. In addition, the selection of variables made by taking previous studies into account and the patients were from a tertiary referral center, potentially contributing to significant bias affecting these statistical analyses. Also, the comparison of the AUC value between different models had certain limitations. Lastly, while a bootstrap testing approach was used to validate our nomogram, the patients used for this validation approach may not be sufficient to ensure the generalizability of these data to patients from other countries or regions. As such, further external validation in a wider pulmonary nodule patient population will be essential in the future.

## Conclusions

In summary, we herein designed a novel nomogram with good accuracy that offers value as a means of differentiating between benign and malignant pulmonary nodules, enabling clinicians to better plan patient treatment. Such individualized risk analyses offer clinicians an opportunity to appropriately monitor and treat patients. However, further work will be needed to validate this nomogram in larger patient populations and to establish whether the treatment decisions made based on this nomogram will reduce rates of incorrect diagnosis and treatment planning for patients with pulmonary nodules.

## Data Availability

All data are fully available from the corresponding author upon reasonable request.

## Abbreviations

LASSO: Least absolute shrinkage and selection operator
NSCLC: Non-small cell lung cancer
BUN: Blood urea nitrogen
GGO: Ground-glass opacity
HPV: Human papillomavirus
HIV: Human immunodeficiency virus
CT: Computed tomography
ROC: Receiver operating characteristic
AUC: Adopting the area under the ROC curve
NRI: Net reclassification improvement index
IDI: Integrated discrimination improvement index
VEGF: Vascular endothelial growth factor
SE: Standard error
